# Transfusion-Related Acute Lung Injury Detected by Perioperative Lung Ultrasound: Early Recognition With Point-of-Care Ultrasound (POCUS)

**DOI:** 10.7759/cureus.93208

**Published:** 2025-09-25

**Authors:** Aaron M Kessler, Arpan Kohli, Grant Neely

**Affiliations:** 1 Anesthesiology, West Virginia University School of Medicine, Morgantown, USA

**Keywords:** acute respiratory distress syndrome (ards), anesthesiology, complications of red blood cell transfusion, critical care, lung ultrasound, point-of-care-ultrasound, transfusion medicine, transfusion-related acute lung injury

## Abstract

Transfusion-related acute lung injury (TRALI) is a rare but potentially life-threatening transfusion reaction characterized by acute hypoxemia and non-cardiogenic pulmonary edema within six hours of transfusion. We report the case of a 20-year-old female patient who developed acute hypoxemia approximately 1.5 hours after receiving one unit of packed red blood cells (PRBCs) during dilation and suction curettage under general anesthesia. Postoperatively, she experienced dyspnea, oxygen desaturation, and coarse bilateral breath sounds. Point-of-care ultrasound (POCUS) demonstrated multiple bilateral B-lines without evidence of pneumothorax, effusion, or cardiac dysfunction, raising suspicion for non-cardiogenic pulmonary edema. Computed tomography (CT) further revealed bilateral ground-glass opacities and septal thickening, supporting the diagnosis of TRALI. The patient required supplemental oxygen and demonstrated rapid clinical improvement. This case highlights the clinical utility of lung POCUS as an early diagnostic adjunct for differentiating TRALI from other causes of acute postoperative respiratory decompensation.

## Introduction

Transfusion-related acute lung injury (TRALI) is a rare but serious complication that occurs within six hours of a blood transfusion, resulting in acute hypoxemia and non-cardiogenic pulmonary edema [1]. The incidence of TRALI in surgical patients who receive blood products is 1.3%-1.4% [1]. The pathogenesis of TRALI is suspected to follow a two-hit model in which the first hit activates pulmonary endothelial cells (ECs) and polymorphonuclear neutrophils (PMNs) [2]. TRALI is classified as Type I (no acute respiratory distress syndrome (ARDS) risk factor) and Type II (with an ARDS risk factor or mild ARDS, provided respiratory status was stable 12 hours prior to transfusion) [3].

Many patient and clinical factors may function as first hits, including hypertension, end-stage liver disease, chronic alcohol use, surgery, mechanical ventilation, trauma, and systemic inflammation [4]. Other pulmonary-related factors that increase risk for TRALI occurrence include pulmonary fibrosis, interstitial lung disease, and tobacco abuse [5]. Transfusion-specific risk factors for the development of TRALI include transfusion of multiple units of blood products and transfusion of plasma from a previously pregnant donor who had developed anti-leukocyte antibodies via alloimmunization [6]. Following the first hit due to these predisposing factors, the second hit is comprised of several mediators in transfused blood products that trigger the primed PMNs and ECs (in addition to monocytes, macrophages, and platelets) to release pathogenic factors and induce coagulopathy, ultimately culminating in intrapulmonary fluid infiltration [2].

Diagnostic characteristics of TRALI include symptom onset within six hours of blood transfusion, oxygen saturation (SpO_2_) < 90% on room air or arterial partial pressure of oxygen (PaO_2_)/fraction of inspired oxygen (FiO_2_) < 300 mmHg on room air, chest X-ray exhibiting evidence of pulmonary edema, risk factors as mentioned above, and brain natriuretic peptide (BNP) < 250 pg/mL [7].

In addition to these diagnostic tools, lung point-of-care ultrasound (POCUS) has been described as of clinical utility for TRALI diagnosis. POCUS rapidly detects interstitial-alveolar syndrome (B-lines) and, combined with basic echocardiography, helps distinguish non-cardiogenic from cardiogenic edema, often outperforming chest radiography for early detection [8]. However, B-lines present on POCUS are not specific for TRALI, and other etiologies of pulmonary edema must be evaluated and excluded. This case report highlights the clinical utility of POCUS as an early diagnostic adjunct tool for the identification of TRALI, in addition to demonstrating the possibility of the development of TRALI in a patient without known preexisting risk factors.

## Case presentation

A 20-year-old female patient (American Society of Anesthesiologists (ASA) I, allergic to penicillins, height 154.9 cm, and weight 57 kg) presented to the hospital with a complaint of severe abdominal pain, which had started one day prior to presentation. The patient endorsed no significant medical history and denied a history of tobacco, drug, or alcohol use, and she was not prescribed any home medications. Additionally, the patient had undergone no prior surgical procedures. Imaging identified fluid-filled endometrium and fluid in the pelvis. The patient was scheduled to undergo dilation and suction curettage under general anesthesia for treatment of suspected hematometra with suspected retrograde menstruation. 

Preoperative laboratory evaluation was significant for anemia and hypokalemia (Table 1), which were not corrected prior to induction of anesthesia. The qualitative serum pregnancy test was negative. The patient's serum antibody screen was negative, and she was crossmatched for two units of packed red blood cells (PRBCs). Preoperative evaluation identified a Mallampati score of 1 with good mouth opening and neck mobility. Cardiac auscultation was insignificant, and breath sounds were clear to auscultation bilaterally. 

**Table 1:** The patient's pertinent laboratory values

Laboratory Measurement	Patient Value	Reference Range	Units
Hemoglobin, preoperative	7.5	11.5-16.0	g/dL
Potassium, preoperative	3.1	3.5-5.1	mmol/L
B-type Natriuretic Peptide (BNP), postoperative	119	< 100	pg/mL

Induction of anesthesia consisted of fentanyl 100 mcg, lidocaine 100 mg, propofol 180 mg, and rocuronium 50 mg. She was intubated with a 7.0 mm endotracheal tube (ETT) using a Macintosh size 3 (MAC 3) blade without difficulty. Sevoflurane was used for maintenance of anesthesia. The patient developed mild hypotension following induction of anesthesia, which was managed with fluid replacement (800 mL over 60 minutes), and she also received 250 mL of 5% albumin for further resuscitation, administered over 15 minutes. Given her starting hemoglobin (Hb) level of 7.5 g/dL and persistent hypotension following crystalloid and colloid resuscitation, one unit (326 mL) of crossmatched non-leukodepleted PRBCs was transfused over five minutes. No other blood products were transfused. Of note, there was no obvious adverse reaction following blood administration. Additionally, due to existing institutional protocols, no donor blood antibody screening was performed prior to blood administration. Anti-HLA testing can be performed per the physician's request prior to administration, but this was not performed for this patient's emergent surgery.

Old blood was identified within the uterus, which was subsequently evacuated. No additional active bleeding was noted. The surgery concluded without complication, and the surgical team reported minimal blood loss (50 mL). A urinary catheter was not placed for this procedure, so no urine output was documented for the surgery. The patient was extubated uneventfully and without evidence of laryngospasm, bronchospasm, or aspiration.

She was transported to the post-anesthesia care unit (PACU) in stable condition. However, within the first hour of her PACU stay, approximately 1.5 hours following completion of the transfusion, she reported increasing dyspnea with oxygen desaturations to approximately 80% on room air. She was given supplemental oxygen via nasal cannula (NC) at 2 liters (L), which improved her SpO_2_ to 90%. This was incrementally increased to 6L NC with subsequent improvement in her oxygenation (SpO₂ 95%-100%). In addition to her respiratory status, the patient was normotensive but tachycardic (heart rate ranging from 102 to 117 beats per minute) throughout this postoperative period. BNP was minimally elevated (Table 1). Unfortunately, no arterial blood gas analysis was completed, which could have aided in ruling out developing ARDS from the differential diagnosis. 

**Figure 1:**
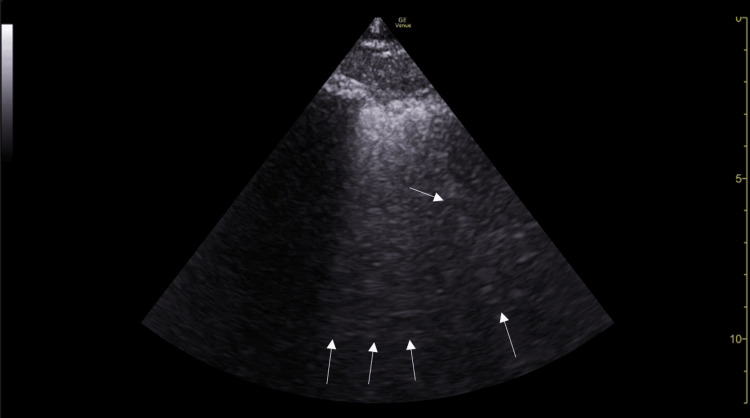
Example lung ultrasound image with multiple B-lines present (white arrows), representative of likely pulmonary edema.

The primary surgical service and supervising anesthesia faculty were notified. Upon evaluation, the patient exhibited no signs of stridor or aspiration. She did display shallow breathing with frequent coughing. Lung auscultation revealed coarse breath sounds bilaterally. The POCUS exam demonstrated no signs of pneumothorax, right heart strain, pleural effusion, or pericardial effusion. However, multiple B lines were appreciated bilaterally on ultrasound in the posterior lower lung fields (Figure 1), suspected to be secondary to pulmonary edema, interstitial lung disease, or ARDS. Of note, no assessment of left ventricular function was documented. This information, if normal, would have further supported the suspected non-cardiogenic etiology of the patient's pulmonary edema, further differentiating TRALI from transfusion-associated circulatory overload (TACO).

**Figure 2:**
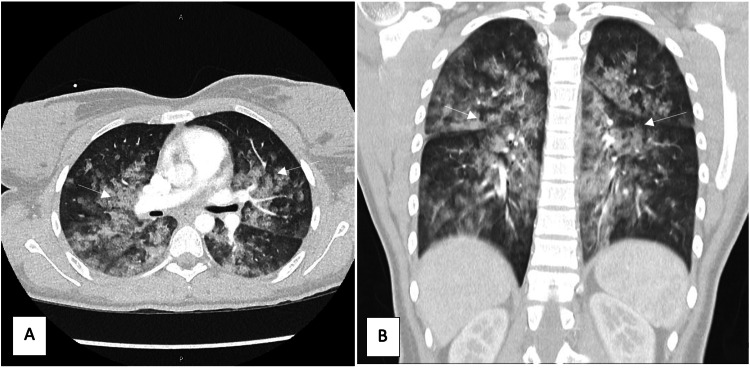
A: Axial cross-section of the chest CT; B: Coronal cross-section of the chest CT Images obtained following admission to the hospital for further observation. Patchy ground-glass opacities are present bilaterally with confluence at the central and hilar regions. Findings are worrisome for alveolar edema.

She was admitted to the hospital for further observation. A computed tomography (CT) scan was completed upon admission (approximately one hour following symptom onset) for further diagnostic clarification and to evaluate for possible pulmonary embolus. The scan identified multiple patchy ground-glass opacities with developing interstitial septal thickening scattered through bilateral lung lobes in a central distribution, concerning for pulmonary edema, further strengthening the suspected diagnosis of TRALI (Figure 2). She required 2L NC overnight with de-escalation to room air the following morning. The patient did not require positive pressure ventilation during her admission. Though not standard treatment for TRALI, she was treated with 40 mg IV furosemide, likely due to confusion surrounding the etiology of the patient's pulmonary edema by the primary admitting medical team. The patient produced a subsequent urine output of 650 mL. The patient was discharged home the following morning. This expedited recovery most likely reflects probable mild Type I TRALI and highlights the necessity for suspicion of TRALI even in patients without preexisting risk factors. 

## Discussion

TRALI is a rare but life-threatening transfusion reaction, defined by the acute onset of hypoxemia and non-cardiogenic pulmonary edema within six hours of transfusion [2,9]. Although incidence is relatively low, TRALI remains a leading cause of transfusion-associated mortality. This case demonstrates a probable presentation of TRALI in a healthy, young patient without preexisting risk factors for the development of TRALI, with an important emphasis on the role of POCUS in early recognition and diagnosis.

Our patient developed dyspnea and hypoxemia within 1.5 hours of transfusion of PRBCs. She exhibited coarse breath sounds, shallow breathing, and hypoxemia refractory to low-flow oxygen, requiring prompt diagnosis and management. A broad differential diagnosis was present, including TRALI, aspiration, bronchospasm, pneumothorax, TACO, or evolving infection. This prompted a unique challenge necessitating early confirmation of diagnosis and initiation of treatment. Given this patient’s adequate nil per os (NPO) timeline prior to induction of anesthesia and lack of gastric contents present in the oropharynx following anesthesia induction and extubation, aspiration was a less favored diagnosis. No documentation or evidence of bronchospasm with the emergence of anesthesia was present, so bronchospasm or complications of bronchospasm were also considered to be less likely diagnoses. No pneumothorax was evident on POCUS as mentioned above. While CT imaging later confirmed bilateral patchy ground-glass opacities consistent with pulmonary edema, the use of bedside ultrasound in the PACU provided rapid and clinically actionable information. Given the lack of BNP elevation, pleural effusion, pericardial effusion, and presence of tachycardia without hypertension, TACO was thought to be much less likely in this clinical setting. 

POCUS revealed multiple bilateral B-lines in the posterior lower lung fields, consistent with interstitial-alveolar syndrome. Despite the patient's lack of risk factors, in the context of recent transfusion, the ultrasound findings favored early suspicion of TRALI over other possible diagnoses as listed above. However, the albumin administration immediately prior to blood transfusion could be considered a priming step to the development of TRALI, but further investigation into this patient, as well as research into this phenomenon, is necessary. Early recognition of symptoms and evaluation with POCUS allowed for timely notification of the surgical and anesthesia teams, initiation of supplemental oxygen, and close monitoring while avoiding unnecessary escalation to mechanical ventilation.

POCUS has increasing value in the perioperative setting as a rapid, repeatable, and noninvasive diagnostic tool. Compared with chest radiography, ultrasound is more sensitive for early identification of pulmonary edema, detecting interstitial changes before other radiographic abnormalities may be apparent [8]. Additionally, the ability to evaluate cardiac function and volume status at the bedside provides a key advantage in distinguishing TRALI from TACO. In this case, the absence of echocardiographic evidence of pericardial or pleural effusions, coupled with the non-cardiogenic pattern of pulmonary edema, reinforced the diagnosis of TRALI.

Management of TRALI largely remains supportive, with oxygen supplementation and, in severe cases, mechanical ventilation. Most patients improve within 48-96 hours, as in our case [9]. Although diuretics were administered empirically, evidence suggests limited benefit since the underlying mechanism is neutrophil-mediated endothelial injury rather than volume overload [2]. Nevertheless, given the diagnostic overlap and clinical commonalities between TRALI and TACO, empiric furosemide use remains common in practice. As with the patient mentioned above, given the improvement noted in this patient's symptoms and high urine output following furosemide administration, consideration should be made for possible concomitant TACO pathophysiology. 

## Conclusions

This case underscores three important lessons: First, TRALI should remain on the differential diagnosis for any patient developing acute hypoxemia within six hours of transfusion. Second, optimization of preoperative anemia via iron or erythropoietin supplementation if appropriate and identification of risk factors for transfusion reactions and transfusion necessity are critical to reduce transfusion requirements, particularly in young, otherwise healthy patients. Finally, and most importantly, this case report highlights the utility of bedside ultrasound as a rapid, non-invasive clinical tool that can expedite diagnosis and guide further management of transfusion-related complications in the perioperative setting.
